# Complications After Maternal Traumatic Brain Injury During Pregnancy: A Systematic Review

**DOI:** 10.1001/jamanetworkopen.2024.59877

**Published:** 2025-02-17

**Authors:** Carina Heller, Mathilda Kraft, Margaret Martinez, Anya S. Mirmajlesi, Magdalena Janecka, Clare McCormack, Moriah E. Thomason, Thomas Weiss, Hector Arciniega

**Affiliations:** 1 Masonic Institute for the Developing Brain, Institute of Child Development, University of Minnesota, Minneapolis; 2 Department of Pediatrics, University of Minnesota, Minneapolis; 3 Department of Psychological and Brain Sciences, University of California, Santa Barbara; 4 Department of Psychiatry and Psychotherapy, Jena University Hospital, Jena, Germany; 5 German Center for Mental Health, Partner Site Jena-Magdeburg-Halle, Jena, Germany; 6 Center for Intervention and Research on Adaptive and Maladaptive Brain Circuits Underlying Mental Health, Partner Site Jena-Magdeburg-Halle, Jena, Germany; 7 Department of Clinical Psychology, Friedrich Schiller University Jena, Jena, Germany; 8 Department of Rehabilitation Medicine, NYU Grossman School of Medicine, New York, New York; 9 NYU Langone Concussion Center, NYU Langone Health, New York, New York; 10 Department of Child and Adolescent Psychiatry, NYU Grossman School of Medicine, New York, New York; 11 Department of Population Health, NYU Grossman School of Medicine, New York, New York

## Abstract

**Importance:**

General trauma is the leading cause of nonobstetric maternal morbidity and mortality, affecting approximately 8% of all pregnancies. Pregnant women with traumatic brain injury (TBI) face high morbidity and mortality rates, requiring complex management due to physiological changes, teratogenic risks of treatments, and the need for fetal monitoring.

**Objectives:**

To assess the consequences of TBI during pregnancy on maternal and fetal outcomes and to evaluate management strategies to inform clinical decision-making.

**Evidence Review:**

A systematic literature search was conducted on January 12, 2024, in PubMed, Web of Science, and PsycInfo to identify articles published in English, German, or Spanish between January 1, 1990, and December 31, 2023, that included at least 1 pregnant individual with TBI. Peer-reviewed, human-based studies with original data on maternal and fetal outcomes were included. Reviews, meta-analyses, and nonhuman studies were excluded. Two independent reviewers screened abstracts and full-text articles. Study characteristics, pregnancy outcomes (maternal and fetal), management methods, and authors’ conclusions were extracted. Risk of bias was assessed by 2 reviewers, with interrater agreement measured using Cohen κ. Disagreements were resolved through discussion. The Preferred Reporting Items for Systematic Reviews and Meta-Analyses (PRISMA) reporting guideline was followed.

**Findings:**

This systematic review included 16 articles involving a total of 4112 individuals (mean maternal age, 26.9 years; range, 16-47 years) who experienced TBI during pregnancy (mean gestational age at injury, 24 weeks; range, 3-38 weeks). The articles comprised 10 case reports, 2 case series, and 4 cohort studies. Motor vehicle crashes were the most common cause of injury, reported in 12 articles. The average Glasgow Coma Scale score ranged from 3 to 15 across all individuals. Conservative management was reported in 7 case patients, whereas surgery was performed in 6 case patients. Maternal outcomes ranged from functional recovery to severe cognitive impairment, and fetal outcomes varied from stable to severe adverse outcomes, including stillbirth and death. Risk of bias assessment indicated moderate to good methodological validity overall, but most articles demonstrated poor quality of evidence.

**Conclusions and Relevance:**

In this review, no definitive association between TBI during pregnancy and maternal or fetal outcomes was found owing to conflicting findings, poor to moderate study quality, and limited evidence. Although some articles suggested increased risks such as placental abruption and cesarean delivery, the findings remained inconclusive. The findings of this review underscore the need for high-quality research, standardized reporting, and rigorous methodology to improve data reliability. Future research should focus on developing consensus-driven, multidisciplinary management strategies to improve maternal and fetal outcomes.

## Key Points

**Question:**

What are the maternal and fetal outcomes of traumatic brain injury (TBI) during pregnancy, and what are the appropriate management strategies?

**Findings:**

This systematic review included 16 articles involving 4112 pregnant individuals with TBI (11 patients from case reports, 10 from case series, and 4091 from cohort studies). No definitive association between TBI and maternal or fetal outcomes was found owing to contradictory findings, poor to moderate quality, and limited evidence.

**Meaning:**

These findings suggest that to provide consensus among management strategies and improve fetal and maternal outcomes, higher-quality research on TBI during pregnancy is urgently needed.

## Teaser

This systematic review examines the association of traumatic brain injury (TBI) during pregnancy with maternal and fetal outcomes.

## Introduction

General trauma is the leading cause of nonobstetric maternal morbidity and mortality, affecting approximately 8% of all pregnancies.^1^ Women have historically been underrepresented in traumatic brain injury (TBI) research,^2,3^ reflecting a broader trend in medical studies.^4^ Retrospective data indicate that the highest percentage of TBI in women occurs between ages 21 and 50 years, encompassing a substantial portion of their reproductive years.^5^ This is concerning because pregnant women with TBI are especially vulnerable^2^ due to maternal physiological adaptations^6^ driven by increased estrogens and progesterone, which are essential for fetal and placental growth.^6,7^ A TBI can disrupt the hypothalamic-pituitary-gonadal axis, affecting these adaptations and potentially affecting both mother and fetus.^8,9^ Consequently, with TBI, there is an increased risk of adverse pregnancy outcomes, such as early membrane rupture, uterine rupture, miscarriage, spontaneous abortion, preterm birth, cesarean delivery, placental abruption, and higher trauma-related mortality.^10,11,12,13,14,15,16^ Managing TBI during pregnancy is complex due to maternal physiological changes,^6,17^ teratogenic risks of pharmacologic therapies, and the need for fetal monitoring.^18^

The elevated morbidity and mortality rates observed in pregnant individuals who sustain a TBI,^15,16^ along with the complexity and increased risk of various adverse outcomes for both mother and fetus,^10,11,12,13,14^ highlight the critical need for an updated and comprehensive analysis of research on TBI during pregnancy. Whereas a previous systematic review^19^ focused solely on case reports and case series, our review expanded on this by including cohort studies. This broader approach provided a more comprehensive analysis of the association of TBI during pregnancy with maternal health, fetal outcomes, and effective management strategies.

## Methods

This systematic review was conducted in accordance with the Preferred Reporting Items for Systematic Reviews and Meta-Analyses (PRISMA) reporting guideline. A preestablished protocol was registered with PROSPERO (CRD42024487480).

### Literature Search

A systematic literature search was performed (by M.K. and M.M.) on January 12, 2024, in the PubMed, Web of Science, and PsycInfo electronic databases. Keywords related to *traumatic brain injury* and *pregnancy* were used. The complete search strategies for all 3 databases, including the number of hits, are detailed in eTable 1 in Supplement 1.

### Eligibility Criteria

Articles were eligible for inclusion if they (1) were published between January 1, 1990, and December 31, 2023; (2) were published in English, German, or Spanish; (3) were human studies; (4) had experimental or epidemiological study designs, including randomized clinical trials, nonrandomized clinical trials, quasi-experimental studies, longitudinal studies, prospective and retrospective cohort studies, cross-sectional studies, case reports, and case series with original empirical data; (5) were peer reviewed; (6) included at least 1 pregnant individual diagnosed with any form of TBI (mild, moderate, or severe) during pregnancy; (7) provided information on maternal outcomes, including TBI severity or maternal mortality; and (8) provided information on fetal outcomes before, during, or after birth (or all), including fetal mortality or adverse birth outcomes (eg, premature birth or low birth weight).

Animal studies, nonhuman studies, and studies without original empirical data (eg, review articles, meta-analyses, and commentaries) were excluded. The complete inclusion and exclusion criteria are detailed in eTable 2 in Supplement 1.

### Literature Screening and Selection Process

Identified articles were manually included in a Mendeley database, with duplicates removed both automatically and manually (Figure). Titles and abstracts of all identified articles were independently screened by 2 reviewers (M.K. and M.M.). Full texts of the remaining articles were further assessed for eligibility. In the case of disagreement, 2 additional reviewers (C.H. and H.A.) provided a secondary assessment. Excluded articles and reasons for exclusion are presented in eTable 3 in Supplement 1.

**Figure.**
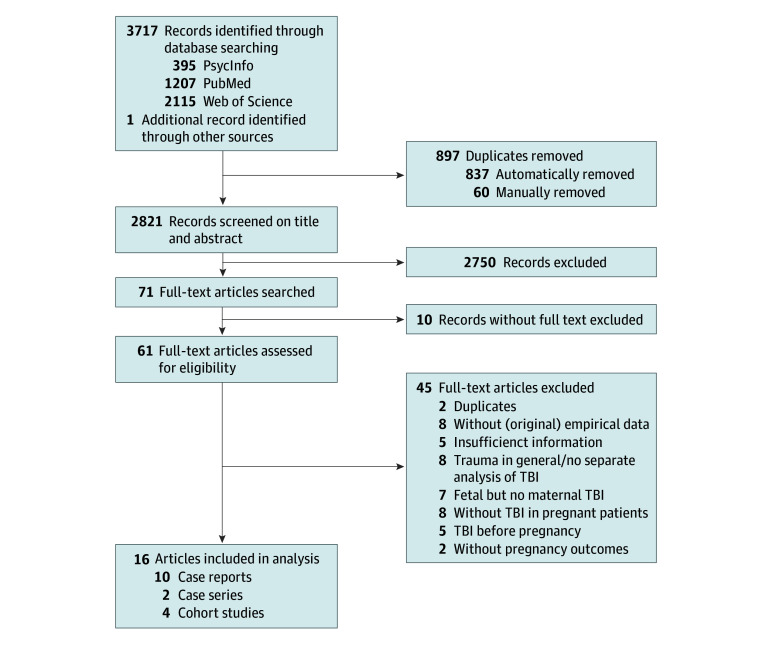
Literature Screening and Study Selection Process TBI indicates traumatic brain injury.

### Data Extraction

Data extraction followed prespecified criteria, including study details, sample characteristics (number of participants, gestational age, mechanism of injury, clinical features, radiographic findings, management methods), and outcomes. Outcomes of interest encompassed information on maternal or fetal conditions during or after pregnancy. Data were extracted by one reviewer (M.K.) and checked by another (M.M.). No meta-analysis was conducted due to the lack of studies with appropriate study design and the high number of case reports.

### Evaluation of Quality of Evidence

The quality of evidence was evaluated using a modified Oxford Centre for Evidence-Based Medicine^20^ rating scheme as follows: (1) properly powered and conducted randomized clinical trial; (2) well-designed controlled trial without randomization or prospective comparative cohort trial; (3) case-control study or retrospective cohort study; (4) case series with or without intervention or cross-sectional study; or (5) opinion of respected authorities or case report. The quality of evidence was initially assessed by one reviewer (M.K.) and verified by another (M.M.).

### Statistical Analysis

Methodological validity was assessed using standardized critical appraisal forms from the Joanna Briggs Institute Meta-Analysis of Statistics Assessment and Review Instrument (JBI-MAStARI).^21^ Interrater agreement for categorical scorings^22^ was assessed with Cohen κ using R, version 4.3.2 (R Project for Statistical Computing), and the DescTools package. *P* < .05 (2-tailed) was considered statistically significant.

## Results

### Included Articles

The electronic database search conducted on January 12, 2024, yielded a total of 3717 records (395 in PsycInfo, 1207 in PubMed, and 2115 in Web of Science) (Figure). Another article, which seemed initially eligible, was identified through a manual search. After title and abstract screening, 61 full-text articles were assessed for eligibility by 2 reviewers (M.K. and M.M.). Forty-five reports were excluded for reasons detailed in the Figure. After full-text screening, a total of 16 articles were eligible and included in the analysis.

### Article Characteristics

Of the 16 articles included in this systematic review, 4 were from the US,^23,24,25,26^ 2 were from Canada,^27,28^ 2 were from India,^29,30^ and 1 article each originated from Taiwan,^31^ Indonesia,^32^ Japan,^33^ France,^34^ Ireland,^35^ Egypt,^36^ Vietnam,^37^ and Finland^38^ (Table 1). The included articles comprised 10 case reports with 11 pregnant patients with TBI,^24,27,28,29,31,32,33,35,36,37^ 2 case series with 10 pregnant patients with TBI,^26,34^ and 4 cohort studies with a total 4091 pregnant patients with TBI.^23,25,30,38^ The 16 included articles involved a total of 4112 individuals experiencing TBI during pregnancy (Table 1).

**Table 1.** Characteristics of Included Articles

Reference, year	Country	Study design	Participants			Gestational age, wk; mechanism of injury	Clinical features	Radiographic findings	Management
			Sample size, No.	Maternal age, y	Preexisting conditions				
Adams et al,^23^ 2023	US	Cohort study	3597 vs 9 106 312 With vs without TBI during pregnancy (N = 9 109 909)	Mean (SD), 27.4 vs 27.8 for patients with TBI at delivery vs without TBI	Psychiatric disorders; substance use disorder; Elixhauser comorbidity index	NA	GCS score: NA; no information	NA	NA
Alley et al,^24^ 2003	US	Case report	1	20	NA	28; MVC	GCS score: 11 (moderate TBI); forehead abrasion; pupils isochoric; normal vital signs	No brain imaging; left diaphragm rupture	Conservative ICU monitoring; no specific medical management identified
Anquist et al,^27^ 1994	Canada	Case report	1	28	Normal pregnancy; hyperemesis	27; MVC (pedestrian)	GCS score: 13 (mild TBI); scalp laceration; transient loss of consciousness, memory loss, disorientation	No brain imaging; multiple extremity fractures	Conservative ICU monitoring; no specific medical management identified
Berry et al, ^25^ 2011	US	Cohort study	71 With TBI during pregnancy vs 8925 nonpregnant female individuals with TBI (N = 18 800)	Mean (SD), 24.9 (7.2) vs 29.0 (10.2) (range, 15-47) for pregnant vs nonpregnant individuals	NA	NA	GCS score: mean (SD), 10.8 (5.0) vs 11.6 (4.7) for pregnant vs nonpregnant individuals; moderate to severe TBI; head AIS score ≥3 (with higher scores indicating more severe injuries)	NA	NA
Chen et al,^31^ 2005	Taiwan	Case report	1	41	No notable past medical history	38; MVC	GCS score: NA; facial lacerations; nausea, vomiting, frontal headache	CT scan: mild DAI with bifrontal traumatic SAH and contusion hemorrhage in the left temporal lobe	ICU monitoring; no specific medical management identified
Darlan et al,^32^ 2021	Indonesia	Case report	1	37	NA	18; MVC	GCS score: 6 (severe TBI); pupils isochoric; right-sided hemiparesis	CT scan: subdural hematoma, burst lobe ICH, SAH, cerebral edema	Primary hematoma-ICH evacuation craniotomy; secondary decompressive craniectomy; precaution measures during intensive care to protect the fetus
			1	31	NA	20; MVC (motorcycle)	GCS score: 6 (severe TBI); pupils isochoric; bilateral decreased light reflexes; decreased light reflexes on both sides	CT scan: multiple contusions, SAH, cerebral edema	Surgery, intracranial pressure monitor; precaution measures during intensive care to protect the fetus
Dawar et al,^29^ 2013	India	Case report	1	26	Full-term uncomplicated pregnancy	36; MVC	GCS score: 11 (moderate TBI); left pupil dilated, nonreactive to light; faciomaxillary injuries	CT scan: acute left frontotemporoparietal SDH with left frontal contusion with midline shift of 5 mm and effaced sulci and left lateral ventricle	Immediate to cesarean delivery decompressive craniectomy; evacuation of SDH, corticectomy, lax duraplasty with pericranial graft
Ganesh et al,^30^ 2022	India	Cohort study	31 Pregnant individuals with TBI vs 259 nonpregnant individuals with TBI (N = 5610)	Mean (SD), 24.2 (3.8) vs 30.3 (7) (range, 18-50) for pregnant vs nonpregnant individuals	NA	NA	GCS score: mean (SD), 11.4 (3.3) vs 11.2 (3.4) (mild, moderate, or severe TBI) for pregnant vs nonpregnant individuals	Rotterdam CT severity (with higher scores indicating more severe injuries): mean (SD), 2.6 (0.8) vs 2.6 (1.0) for pregnant vs nonpregnant individuals	Surgical intervention for 8 vs 50 pregnant vs nonpregnant individuals (*P* = .60)
Hnat et al,^28^ 2003	Canada	Case report	1	23 or 28 (Text vs abstract)	NA	3; MVC	GCS score: 4 (severe TBI); hypotensive	CT scan: diffuse cerebral edema with obliteration of the basilar cistern; closed head injury; liver laceration	Primary ICU management with pressors, sedation and intracranial pressure control with mannitol; secondary ventriculostomy
Inoue et al,^33^ 2015	Japan	Case report	1	16	NA	11; MVA (motorcycle)	GCS score: 5 (severe TBI); normal vital signs; prolonged loss of consciousness; decerebrate posturing; anisocoria; abnormal eye reflexes	(1) CT scan: left temporal lobe and corpus callosum contusion, SAH, DAI; (2) MRI scan: diffuse petechial hemorrhage in the basal temporal lobe	Primary ICU monitoring without specific medical management specified
Kissinger et al,^26^ 1991	US	Case series	93 (9 With severe head injury during pregnancy)	Mean, 24.8 (range, 15-42)	NA	Mean, 21.9; MVC (n = 63), gunshot (n = 6), stab wound (n = 2), fall (n = 9), blunt assault (n = 11), burn (n = 1), or other (n = 2)	GCS score: mean, 12 vs 14.5 in nonviable vs viable pregnancies; ISS: mean, 8.5 (range, 1-75)	Diagnostic radiography (n = 69); pelvic ultrasonography (n = 67); head CT scan (n = 32); abdominal/pelvic CT scan (n = 16); diagnostic peritoneal lavage (n = 6)	Cardiotocographic monitoring (n = 39); nonobstetrical surgical procedures (n = 23)
Leroy-Malherbe et al,^34^ 2006	France	Case series	18 (1 With brain injury during pregnancy)	NA	NA	29; MVC	GCS score: NA; brain injury, coma for 1.5 mo	NA	NA
Neville et al,^35^ 2012	Ireland	Case report	1	27	Recurrent early pregnancy loss; epilepsy; severe postnatal depression	13; MVC	GCS score: 6 (severe TBI)	CT scan: hemorrhagic contusions, diffuse brain swelling, multiple skull and facial fractures; cerebral edema, traumatic skull fracture, temporal contusions	Primary intracranial pressure monitoring; secondary decompressive craniectomy; ultrasounds for monitoring fetal well-being
Tawfik et al,^36^ 2015	Egypt	Case report	1	27	No notable past medical history	37; Blunt injury (fallen object)	GCS score: 11 (moderate TBI); left pupil dilated, nonreactive to light; left temporal laceration	CT scan: depressed left temporal bone fracture, large acute left temporoparietal epidural hematoma	Craniotomy and evacuation of the epidural hematoma
Tran et al,^37^ 2021	Vietnam	Case report	1	20	No notable past medical history	30; MVC (motorcycle)	GCS score: 3 (severe TBI); pupils dilated and nonresponsive; coma; otherwise, vitals within normal limits	CT: large extradural hematoma in the right temporal-parietal-occipital region	Tracheal intubation; emergency extradural hematoma evacuation with craniectomy
Vaajala et al,^38^ 2023	Finland	Cohort study	392 With vs 1176 without TBI during pregnancy (matched reference group)	Mean (SD), 28.5 (6.5) vs 28.5 (5.9) (range, 15-49) for individuals with vs without TBI during pregnancy	NA	NA	GCS score: NA; no information	Concussion (n = 359 [91.6%]); diffuse TBI (n = 11 [2.8%]); traumatic subdural hemorrhage (n = 7 [1.8%]); unspecified intracranial injury (n = 6 [1.5%])	NA

Abbreviations: AIS, Abbreviated Injury Scale; CT, computed tomography; DAI, diffuse axonal injury; GCS, Glasgow Coma Scale; ICH, intracerebral hemorrhage; ICU, intensive care unit; ISS, injury severity score; MVC, motor vehicle crash; NA, not available; SAH, subarachnoid hemorrhage; SDH, subdural hemorrhage; TBI, traumatic brain injury.

The mean maternal age of pregnant individuals with TBI was 26.9 years (range, 16-47 years), although 2 articles did not report or provide missing or inconsistent information on maternal age.^28,34^ Nine articles did not include information on maternal preexisting conditions.^24,25,26,28,30,32,33,34,38^ However, 2 articles listed previous pregnancy complications or disorders,^23,35^ and 5 articles reported a normal medical history, normal current pregnancy, or both.^27,29,31,36,37^ The mean gestational age at injury was 24 weeks (range, 3-38 weeks), with 4 articles lacking this information.^23,25,30,38^ Motor vehicle crashes were the most common cause of injury, reported in 74 case patients.^24,26,27,28,29,31,32,33,34,35,37^ The average Glasgow Coma Scale (GCS)^39^ score across all case reports was 7.6, encompassing 1 case patient with mild TBI,^27^ 3 with moderate TBI,^24,29,36^ and 6 with severe TBI^28,32,33,35,37^ as detailed in the individual case reports. In 1 case series, the average GCS score was 13.2, with viable pregnancies having a GCS score of 14.5 and nonviable pregnancies having a GCS score of 12.^26^ Additionally, 2 cohort studies, which included patients with mild, moderate, and severe TBI, reported average GCS scores of 10.8 and 11.4, respectively.^25,30^ Four articles did not report GCS scores or TBI severity.^23,31,34,38^

Radiographic findings using computed tomography (CT) included contusions in 5 case patients,^29,31,32,33,35^ subarachnoid hemorrhage in 4 case patients,^31,32,33^ cerebral edema in 4 case patients,^28,32,35^ hematoma in 3 case patients,^31,36,37^ diffuse axonal injury in 2 case patients,^31,33^ subdural hemorrhage in 1 case patient,^29^ and intracerebral hemorrhage in 1 case patient^32^ and were reported in 8 case reports.^28,29,31,32,33,35,36,37^ In 8 of the case patients, multiple findings occurred simultaneously.^28,29,31,32,33,35,36^ Other findings included rupture of the diaphragm without brain imaging in 1 case patient^24^ and scalp laceration with a closed head injury in 1 case patient^27^; information on Rotterdam CT severity was provided in 1 cohort study,^30^ and various diagnostic methods were described in 1 case series^26^ and in 1 cohort study.^38^ Three articles did not provide information on radiographic findings.^23,25,34^ Regarding treatment methods, conservative treatment was reported in 7 case patients.^24,27,28,31,32,33,35^ Surgical procedures, including ventriculostomy, hematoma evacuation, craniotomy, or craniectomy, were performed in 6 case patients.^28,29,32,35,36,37^ Four articles did not report management methods (Table 1).^23,25,34,38^

### Risk of Bias Assessment

Most articles included in this systematic review provided poor-quality evidence: 10 case reports^24,27,28,29,31,32,33,35,36,37^ were rated 5 and 2 case series^26,34^ were rated 4. Four cohort studies were rated 3, indicating a moderate quality of evidence.^23,25,30,38^ The methodological validity of all included articles was found to be at least moderate (Table 2). Detailed critical appraisal using JBI-MAStARI criteria is presented in eTable 4 in Supplement 1. The critical appraisal conducted by 2 raters (M.K. and M.M.) yielded a κ of 0.76 (95% CI, 0.65-0.87), indicating substantial interrater reliability. Missing data, due to various reasons, in participant characteristics, clinical features, radiographic findings, management methods, and maternal or fetal outcomes of individual articles resulted in incomplete information.

**Table 2.** Results of Included Articles

Reference, year	Pregnancy outcome^a^			Risk of bias		Conclusion and suggestions
	General	Maternal	Fetal	Rating, quality of evidence^b^	Score, methodological validity^c^	
Adams et al,^23^ 2023	Pregnancy complications: gestational diabetes (RR, 0.91 [95% CI, 0.79-1.04]); preeclampsia and eclampsia (95% CI, 0.88 [0.75-1.05]); placenta previa (95% CI, 0.79 [0.49-1.30]); ↑ placental abruption (95% CI, 2.73 [2.26-3.30]); labor and delivery outcomes: breech (95% CI, 0.97 [0.81-1.18]); ↑ cesarean delivery (95% CI, 1.83 [1.75-1.91]); ↑ antepartum hemorrhage (95% CI, 2.32 [1.95-2.75]); postpartum hemorrhage (95% CI, 0.98 [0.81-1.19])	GOS score: NA; no information	Preterm birth (RR, 1.10 [95% CI, 0.98-1.24]); ↑ stillbirth (2.55 [1.97-3.29]); small for gestational age (0.99 [0.80-1.23]); ↑ large for gestational age (1.30 [1.09-1.56])	3, Moderate	8/11, Moderate	(1) Potential pregnancy complications for women with TBI; (2) closer monitoring to optimize maternal and fetal outcomes; (3) routine screening for TBI during preconception and early in pregnancy
Alley et al,^24^ 2003	Concealed placental abruption; emergent, low transverse cesarean delivery	GOS score: NA; no information	No heart rate at delivery (resuscitation); ischemia and brain injury: massive bilateral subdural hematomas; diffuse decreased density of cerebral and cerebellar parenchyma with loss of gray/white differentiation suggestive of diffuse cerebral edema; birth weight: 1355 g; length: 41 cm; AS at 1 min: 1; AS at 5 min: 3; AS at 10 min: 3; death 20 h after delivery	5, Poor	6/8, Moderate	(1) Fetal assessment after maternal injury; (2) screening ultrasound should be performed by radiology/neonatology to look for intracranial fetal injury; (3) neurosurgery should be on standby to perform emergent craniotomy if survivable intracranial injury is identified in the fetus
Anquist et al,^27^ 1994	Normal placenta without evidence of separation directly after injury; full-term pregnancy at 37 wk of gestation; small placenta with diffuse calcification after delivery	GOS score: NA; stable condition 2 d after accident; residual disorientation because of head injury; discharged at HD 2	Fetal growth retardation; birth weight: 2300 g; AS at 1 min: 4; AS at 5 min: 7; 2 documented seizures; EEG: severe background abnormalities with right hemispheric epileptiform activity; ultrasound: dilated ventricles and cystic areas in the frontal regions of the brain; CT scan (after injury): no evidence for a fetal skull fracture; CT scan (after delivery): irregular multifocal hypodensities, calcification, moderate bilateral ventriculomegaly; follow-up (4 y): cortically blind, with epilepsy, severe spastic quadriparesis	5, Poor	5/8, Moderate	(1) Differences between fetal assessment at time of injury from fetal outcomes at birth and follow-up; (2) accurate fetal assessment may help obstetricians to decide whether aggressive intervention is needed for saving the fetus in the immediate posttraumatic period; (3) evaluation of fetal structural integrity of the cerebrum with transvaginal ultrasound or MRI
Berry et al,^25^ 2011	NA	GOS score: NA; patients aged 15-47 y: similar mortality rates in pregnant and nonpregnant patients with TBI (9.9% vs 6.8%; *P* = .34); (3) ↑ mortality (AOR, 2.0 [95% CI, 0.8-4.6]; *P* = .12) after adjusting for confounding variables	NA	3, Moderate	8/11, Moderate	(1) Pregnant patients with moderate to severe TBI without statistically significant difference in mortality when compared with nonpregnant counterparts; (2) after adjusting for confounding variables, higher mortality observed in the pregnant group: no neuroprotective effect attributed to female sex hormones
Chen et al,^31^ 2005	Vaginal delivery; no complications	GOS score: NA; discharged at HD 7	Healthy infant; birth weight: 3525 g	5, Poor	7/8, High	(1) Advantages and disadvantages of anesthetic techniques for head-injured patients in labor should be carefully weighed against each other; (2) continuous lumbar epidural analgesia with 0.2% ropivacaine as a sound alternative
Darlan et al,^32^ 2021	Viable pregnancy	GOS score: 10; after surgery: isochoric pupils, reactive to light, improved hemiparesis; CT scan: no sign of SDH-ICH, midline shift disappeared, remaining cerebral edema	Stable fetal condition; no sign of fetal distress	5, Poor	6/8, Moderate	(1) Physiological changes in pregnancy with an important role in the management of TBI in pregnancy; (2) saving the maternal-fetal condition as main goal for management of TBI in pregnancy; (3) gestational age as an important factor in choosing timing of surgery (neurosurgery and obstetric procedures); (4) multidisciplinary discussion
	Viable pregnancy	GOS score: NA; no information	No sign of fetal distress; maintaining fetal condition without obstetric intervention after ICP monitoring	5, Poor	5/8, Moderate	
Dawar et al,^29^ 2013	Cesarean delivery	GOS score: 15; discharged at HD 10; 1-y follow-up: no neurological deficits	Fetal distress; healthy infant; birth weight: 2980 g; AS at 1 min: 6; AS at 5 min: 9; 1-y follow-up: no neurological deficits	5, Poor	6/8, Moderate	(1) Care of the pregnant neurosurgical patient follows the general principles of obstetrics and neurosurgery with some specific considerations; (2) simultaneous craniotomy and cesarean delivery in pregnant patients with moderate to severe head injury using a multidisciplinary team approach
Ganesh et al,^30^ 2022	No correlation of gestational age with severity of head injury (*P* = .54) and with outcome (*P* = .36)	GOS score: mean (SD) 4.80 (0.59) vs 4.13 (1.58) for pregnant vs not pregnant individuals (*P* = .02)	NA	3, Moderate	6/11, Moderate	(1) Variable management and outcomes of TBI in pregnancy; (2) potentially better 3-mo outcomes among pregnant patients with TBI; (3) potential improvement in neurological outcome and survival benefit of pregnant patients with TBI in the acute phase of injury
Hnat et al,^28^ 2003	No intrapartum complications; vaginal delivery on 36 to 37 wk of gestation	GOS score: NA; discharged at HD 242; follow-up (1 y): major neurological deficits	Healthy infant; birth weight: 3363 g; AS at 1 min: 9; AS at 5 min: 9; follow-up (1 y): normal development, no neurological complications	5, Poor	6/8, Moderate	(1) Management of TBI during pregnancy with a multidisciplinary approach, including collaboration with health care professionals and family members; (2) consider the prognosis of mother and fetus along with the gestational age of the fetus in decision-making
Inoue et al,^33^ 2015	Dilation and curettage at HD 51	GOS score: NA; PSH diagnosis at HD 10; discharged at HD 117	Abdominal ultrasound: no evidence of fetal damage; fetus in good health until day 25; IUFD after 16 wk of gestation (HD 37)	5, Poor	7/8, High	(1) Pregnancy and IUFD as potential risk factors for PSH exacerbation; (2) delivery can be useful to resolve refractory PSH and save the mother’s life
Kissinger et al,^26^ 1991	Cesarean delivery (n = 5)	GOS score: NA; 3 maternal deaths (3%); discharged at HD (mean, 7.1; range, 1-76)	(1) 14 Deaths (15%) (8 fetal deaths, 4 elective abortions; and 2 neonatal deaths); and (2) 39 with long-term follow-up (21 alive and 18 deaths)	4, Poor	10/10, High	(1) Direct injuries to the uteroplacental fetal unit and maternal death, hypoxia, shock, pelvic fracture, and severe head injury identified as conditions associated with increased risks of fetal death; (2) additional monitoring techniques may be required
Leroy-Malherbe et al,^34^ 2006	Emergency cesarean delivery at 29 gestational wk; microthrombi on the placenta	GOS score: NA; discharged after 6 mo hospitalization	No reactive cardiac rhythm (immediately); birth weight: 1240 g; AS at 1 min: 6; AS at 5 min: 7; AS at 10 min: 8; atrophy of the posterior part of corpus callosum; follow-up (4 y): autistic symptoms; global backwardness (GOS score = 3)	4, Poor	6/10, Moderate	(1) Maternal trauma as one of the environmental factors responsible for cerebral palsy and its complications; (2) fetal injury comes from a wide range of mechanisms; (3) posttraumatic evaluation of the fetus often incomplete or absent
Neville et al,^35^ 2012	Delayed cesarean delivery at a mean (SD) of 35 (3) gestational wk	GOS score: NA; follow-up (8 mo): severe cognitive impairment; Disability Rating Scale score: 21 of 29, indicating extremely severe disability	Healthy infant; birth weight: 2770 g	5, Poor	7/8, High	(1) Neurotrauma in pregnancy with management in a tertiary care center with multidisciplinary input; (2) aggressive initial neurosurgical treatment proven beneficial for optimal management of fetus and mother; (3) long-term care of patients with TBI in this circumstance; (4) anticipation of the needs of both the child and the mother
Tawfik et al,^36^ 2015	No signs of placental abnormalities; cesarean delivery followed by craniotomy	GOS score: NA; discharged at HD 4	No signs of fetal distress; healthy infant; birth weight: 3200 g; AS at 1 min: 8; AS at 5 min: 10	5, Poor	8/8, High	(1) Cesarean delivery followed by decompressive craniotomy under general anesthesia; (2) management of pregnant patients with TBI with a multidisciplinary team for the best outcome of the mother and her fetus
Tran et al,^37^ 2021	Cesarean delivery	GOS score: 15; discharged at HD 21; follow-up (7 wk): no neurological deficits; follow-up (23 wk): no neurological deficits, spontaneous cranial bone regeneration	Healthy infant; no further information	5, Poor	7/8, High	(1) Transfer pregnant patients with possible head trauma to a center with expertise in neurotrauma and obstetrical care; (2) surgery techniques and hormones in pregnancy with potential contribution to bone formation
Vaajala et al,^38^ 2023	↑ Cesarean delivery in women with TBI (21.4% vs 15.5%; *P* = .008), specifically in women with TBI during third trimester (29.0% vs 15.0%; *P* = .02); ↑ labor induction in women with TBI during first trimester (24.2% [95% CI, 17.9%-32.1%] vs 19.2% [95% CI, 15.8%-23.1%]); ↓ spontaneous vaginal birth (66.1% [95% CI, 58.3%-74.6%] vs 75.6% [95% CI, 71.0%-81.0%), ↑ assisted vaginal birth (12.5% [95% CI, 9.3%-16.5%] vs 8.7% [95% CI, 7.1%-10.5%]), ↑ elective cesarean delivery (9.7% [95% CI, 6.9%-13.3%] vs 6.5% [95% CI, 5.1%-8.1%]); no difference in uterine curettage (<1.3% vs 0.6%; *P* = .68) or manual placenta removal (<1.3% vs 0.2%; *P* = .58)	GOS score: NA; no information	Preterm birth (<37 wk) (5.4% vs 4.7%; *P* = .74); low birth weight (<2500 g) (4.8% vs 2.6%; *P* = .05); perinatal mortality (<1.3% vs <0.4%; *P* > .99); ICU (12% vs 10.9%; *P* = .61)	3, Moderate	8/11, Moderate	(1) TBI during pregnancy associated with increased rate for cesarean delivery; (2) no evidence for difference in fetal outcomes (preterm birth, low birth weight, need for ICU); (3) mild TBIs during pregnancy with fewer adverse outcomes on fetal health

Abbreviations: AOR, adjusted odds ratio; AS, Apgar score; CT, computed tomography; EEG, electroencephalogram; GOS, Glasgow Outcome Scale; HD, hospital day; ICH, intracerebral hemorrhage; ICP, intracranial pressure; ICU, intensive care unit; IUFD, intrauterine fetal death; JBI-MAStARI, Joanna Briggs Institute Meta-Analysis of Statistics Assessment and Review Instrument; MRI, magnetic resonance imaging; NA, not available; PSH, paroxysmal sympathetic hyperactivity; RR, relative risk; SDH, subdural hemorrhage; TBI, traumatic brain injury.

^a^ Upward and downward arrows indicate increased and decreased risk for pregnant patients with TBI, respectively.

^b^ Quality of evidence was assessed using a rating scheme modified from the Oxford Centre for Evidence-Based Medicine^20^ for ratings of individual studies.

^c^ Methodological validity was evaluated using JBI-MAStARI standardized critical appraisal assessment forms.^21^ Further details are provided in eTable 4 in Supplement 1.

### Pregnancy Outcomes

Fourteen articles provided information on pregnancy outcomes.^23,24,26,27,28,29,31,32,33,34,35,36,37,38^ The remaining 2 articles did not provide this information.^25,30^

#### Case Reports and Series

Ten case reports^24,27,28,29,31,32,33,35,36,37^ and 2 case series^26,34^ provided information on pregnancy outcomes. Five case reports documented cesarean birth as the delivery method following maternal TBI,^29,34,35,36,37^ and 1 article reported concealed placental abruption.^24^

#### Cohort Studies

Two cohort studies provided information on pregnancy outcomes.^23,38^ In their cohort study, Adams et al^23^ found an increased risk of placental abruption (relative risk [RR], 2.73 [95% CI, 2.26-3.30]); associated sequelae, such as antepartum hemorrhage (RR, 2.32 [95% CI, 1.95-2.75]); and cesarean delivery (RR, 1.83 [95% CI, 1.75-1.91]) when comparing 3597 deliveries of women with TBI to 9 106 312 deliveries of women without a TBI diagnosis. The cohort study by Vaajala et al,^38^ which included 392 women with TBI during pregnancy and a matched group of 1176 women (from a control group of 722 497 women) without TBI during pregnancy, suggested a higher rate of cesarean delivery among the TBI group compared with their matched counterparts (21.4% vs 15.5%; *P* = .008). Women who experienced TBI during the third trimester had an even higher rate of cesarean delivery (29.0% vs 15.0%; *P* = .02) (Table 2).

### Maternal Outcomes

Thirteen articles provided information on maternal outcomes.^25,26,27,28,29,30,31,32,33,34,35,36,37^ The remaining 3 articles did not provide this information.^23,24,38^

#### Case Reports and Series

No maternal abnormalities following TBI management were observed in 7 case patients.^27,29,31,32,34,36,37^ Adverse maternal outcomes, including major neurological deficits,^28^ severe cognitive impairment,^35^ as well as pregnancy-induced and intrauterine fetal death–induced exacerbation of paroxysmal sympathetic hyperactivity,^33^ were reported in 3 case patients. Tran et al^37^ observed a spontaneous cranial bone regeneration in their pregnant patient with TBI and concluded that pregnancy hormones may contribute to bone formation.

#### Cohort Studies

In their cohort study, Ganesh et al^30^ found better functional 3-month outcomes when comparing Glasgow Outcome Scale (GOS) scores of 31 pregnant patients with TBI (mean [SD], 4.80 [0.59]) and 228 nonpregnant patients with TBI (mean [SD], 4.13 [1.58]) (*P* = .02). The authors suggested a possibility of improved neurological outcomes and survival benefits for pregnant patients with TBI in the acute phase of injury, suggesting protective effects of pregnancy in patients with TBI.^30^ However, Berry et al^25^ found similar mortality rates in pregnant and nonpregnant patients with TBI (9.9% vs 6.8%; *P* = .34) in the subgroup analysis of patients with TBI aged 15 to 47 years (n = 8854). It should be noted that Berry et al^25^ observed that mortality among pregnant patients with TBI had an adjusted odds ratio greater than 1, but this was not significant (adjusted odds ratio, 2.0 [95% CI, 0.8-4.6]; *P* = .12) after adjustment for confounding factors (eg, age, systolic blood pressure, injury severity score, Abbreviated Injury Scale [AIS] score,^40^ GCS score, and outcome such as any complication, hospital and intensive care unit length of stay, and mortality). Berry et al^25^ concluded there was no neuroprotective effect of female sex hormones.

### Fetal Outcomes

Fourteen articles reported information on fetal outcomes.^23,24,26,27,28,29,31,32,33,34,35,36,37,38^ Two articles did not provide this information.^25,30^

#### Case Reports and Series

In 7 case reports, stable fetal conditions, no signs of fetal distress, or delivery of a healthy infant despite a maternal TBI diagnosis were reported.^28,29,31,32,35,36,37^ However, in other cases, adverse fetal outcomes were observed, including intrauterine fetal death,^33^ consequent fetal death 20 hours after delivery,^24^ fetal seizures and blindness as well as epilepsy in the 4-year follow-up,^27^ and no cardiac rhythm, autistic symptoms, and global retardation 4 years later.^34^ In the case series by Kissinger et al^26^ of 93 pregnant patients with TBI, 14 deaths were observed, including 8 fetal deaths (2 associated with maternal death), 4 cases of elective abortion, and 2 neonatal deaths. Of the 9 patients with severe head injury, defined as a head injury score of 3 or higher on the AIS scale,^40^ 5 subsequently experienced fetal loss.^26^

#### Cohort Studies

In their cohort study, Adams et al^23^ reported an increased risk of stillbirth (RR, 2.55 [95% CI, 1.97-3.29]) and delivery of large for gestational age infants (RR, 1.30 [95% CI, 1.09-1.56]) for women with TBI. However, Vaajala et al^38^ reported that more infants born to individuals with TBI during pregnancy compared with those without TBI had low birth weight, although the findings were not statistically significant (4.8% vs 2.6%; *P* = .05).

## Discussion

This review highlights the variability in TBI outcomes among pregnant individuals, underscoring the critical need to address research gaps and establish consensus on tailored clinical management strategies. A spectrum of pregnancy, maternal, and fetal outcomes, with evidence predominately from poor-quality case reports and moderate-quality cohort studies, was observed. Notably, although some cohort studies suggested an association between TBI and adverse pregnancy outcomes, such as increased rates of placental abruption, cesarean delivery, and low birth weight, definitive conclusions remain elusive due to inconsistencies in study design and data quality.

A total of 16 articles were included in this review (4 of which were cohort studies,^23,25,30,38^), encompassing 4112 pregnant patients with TBI. The risk of bias assessment suggested that most articles provided poor-quality or moderate-quality evidence on TBI during pregnancy. Case reports^24,27,28,29,31,32,33,35,36,37^ and case series,^26,34^ which dominated the included articles, offered lower levels of evidence on the consequences of TBI during pregnancy owing to their descriptive nature and small sample sizes, reflecting the opportunistic nature of most studies in this field. The 4 cohort studies also exhibited moderate quality of evidence.^23,25,30,38^ Furthermore, the results suggest gaps and inconsistencies in reporting, particularly regarding maternal preexisting conditions, gestational age at injury, and radiographic findings. This variability emphasizes the need for standardized reporting criteria in case reports, case series, and cohort studies to enhance data comparability and reliability.

There was no clear association between TBI and adverse pregnancy outcomes based on the results of this review. However, in their cohort study, Adams et al^23^ found a higher risk of placental abruption and cesarean delivery among pregnant women with TBI compared with those without TBI. Additionally, the cohort study by Vaajala et al^38^ indicated an elevated rate of cesarean deliveries, particularly following TBI in the third trimester. The findings of these cohort studies align with previous studies that have reported similar risks,^10,11,12,13^ suggesting that TBI can substantially affect pregnancy outcomes.

Maternal outcomes following TBI varied in this review. Some articles reported no substantial abnormalities,^27,29,31,32,34,36,37^ whereas others noted severe complications such as major neurological deficits^28^ and cognitive impairments.^35^ Interestingly, Ganesh et al^30^ reported better 3-month outcomes among pregnant patients with TBI, suggesting potential neuroprotective effects of pregnancy-related hormones in both the acute and rehabilitative phases of head injury. However, these findings are not universally supported, as documented by inconsistent results regarding the neuroprotective role of progesterone in TBI recovery.^41,42,43,44,45^ Furthermore, Berry et al^25^ reported no statistically significant difference in mortality between pregnant and nonpregnant women with TBI after adjusting for confounding variables, although a nonsignificant trend toward increased mortality in pregnant patients with TBI was noted.

Fetal outcomes also demonstrated variability across the articles in this review. Although some articles reported good fetal functioning following invasive maternal management, even in cases of severe maternal injury or brain death,^28,32,35,46^ adverse outcomes such as preterm birth, stillbirth, subsequent death after delivery, and adverse follow-up outcomes have also been documented.^23,24,27,33,34^ The increased risk of stillbirth and large for gestational age infants, as reported by Adams et al,^23^ and low birth weight infants, as noted by Vaajala et al,^38^ underline the complexity and heterogeneity of fetal outcomes following maternal TBI. These findings highlight the need for close fetal monitoring and neonatal care for infants delivered by mothers with acquired TBI during pregnancy. The ethical considerations surrounding balancing invasive maternal treatment with expected maternal and fetal quality of life are complex and require careful deliberation.^47^ In a 2021 preclinical study, Saber et al^48^ suggested that maternal TBI may have lasting effects on offspring, including disrupted anxiety-like behavior and impaired neural connectivity. The full range of health concerns and quality of life for neonates born to women who experienced TBI during pregnancy are not fully understood. The results of the included articles suggest the need for ongoing research into the long-term consequences of maternal TBI on the offspring.

Among the included articles, management approaches for TBI during pregnancy were often conservative,^24,27,28,31,32,33,35^ but surgical interventions, such as hematoma evacuation and craniotomy, were required in severe cases.^28,29,32,35,36,37^ Protective measures for the fetus during intensive care for pregnant patients with TBI included intracranial pressure monitoring and, in urgent cases, cesarean delivery followed by craniectomy.^29,35^ Gestational age influences the timing of both neurosurgical and obstetrical procedures.^28,32^ Therefore, a multidisciplinary discussion involving neurosurgeons, obstetricians, neonatologists, and anesthetists is recommended for managing TBI in pregnancy.^28,29,32,35,36^ Delayed recognition of fetal distress and delayed cesarean delivery can lead to fetal deaths.^49^ Consequently, pregnant trauma patients and their (unborn) children should be monitored from the time of injury.^23,24,26,27,50^

Algorithms used to manage adult patients with TBI are not fully applicable to pregnant patients due to their exclusion from most TBI studies. These factors lead to challenges in applying general management principles.^18,29^ The variability in radiographic findings and treatment approaches among the included articles emphasizes the need for individualized pregnancy-specific guidelines. Additionally, collecting more detailed data to improve treatment guidelines for this unique population is crucial and currently lacking. Di Filippo et al^51^ provided management suggestions for moderate to severe TBI in pregnant women, considering the pathophysiologic changes of pregnancy and differences in specific parameters such as cerebral perfusion pressure and arterial carbon dioxide pressure targets. However, there are currently no management guidelines for mild TBI during pregnancy, despite its potential to cause physiological changes that could adversely affect both the mother and the (unborn) child.^27,52^

### Limitations

This review has some limitations. The limited generalizability of the findings stems from the moderate to poor quality of evidence in the included articles, often characterized by small sample sizes; inconsistent data on clinical features and outcomes of TBI during pregnancy, including radiologic findings and TBI severity indices such as GCS; as well as a lack of appropriate control groups in most articles. Many articles focused on immediate short-term outcomes, resulting in a gap in knowledge regarding the long-term outcomes for children born to mothers with acquired TBI during pregnancy and for the women themselves. Despite these limitations, this systematic review provides valuable insights into TBI during pregnancy, extending beyond case reports and case series by including 4 cohort studies.^23,25,30,38^

## Conclusions

In this systematic review, no definitive association between TBI and maternal or fetal outcomes was found owing to conflicting findings, poor to moderate study quality, and limited evidence. These findings emphasize the critical need for tailored clinical management strategies for TBI during pregnancy, considering the complexity and variability of pregnancy, maternal, and fetal outcomes. Future research should prioritize larger, high-quality studies that provide comprehensive data on both short-term and long-term outcomes for mothers and their children. Prospective and longitudinal studies are needed to understand better the mechanisms underlying adverse outcomes and to identify potential protective factors. These results serve as a call to action for higher-quality research and improved data reliability to foster consensus on multidisciplinary care, fetal monitoring, and standardized reporting.

## Notes

Supplement 1. **eTable 1.** Search Strategy for PubMed, Web of Science, and PsycInfo With Number of Hits (January 12, 2024) **eTable 2.** Inclusion and Exclusion Criteria for Selection of Relevant Studies **eTable 3.** Excluded Studies, With Reasons for Exclusion **eTable 4.** Critical Appraisal With Criteria From the Joanna Briggs Institute Meta-Analysis of Statistics Assessment and Review Instrument (JBI-MAStARI) for Case Reports

Supplement 2.
Data Sharing Statement
