# Supplementary material for: Complications After Maternal Traumatic Brain Injury During Pregnancy: A Systematic Review
Source: JAMA Netw Open. 2025 Feb 17;8(2):e2459877. doi: 10.1001/jamanetworkopen.2024.59877 (PMC11833521; doi:10.1001/jamanetworkopen.2024.59877)
Supplement: Supplement 1. — eTable 1. Search Strategy for PubMed, Web of Science, and PsycInfo With Number of Hits (January 12, 2024) eTable 2. Inclusion and Exclusion Criteria for Selection of Relevant Studies eTable 3. Excluded Studies, With Reasons for Exclusion eTable 4. Critical Appraisal With Criteria From the Joanna Briggs Institute Meta-Analysis of Statistics Assessment and Review Instrument (JBI-MAStARI) for Case Reports [file jamanetwopen-e2459877-s001.pdf]

Supplementary Online Content

Heller C, Kraft M, Martinez M, et al. Complications after maternal traumatic brain injury during pregnancy: a systematic review. *JAMA Netw Open*. 2025;8(2):e2459877. doi:10.1001/jamanetworkopen.2024.59877

eTable 1. Search Strategy for PubMed, Web of Science, and PsycInfo With Number of Hits (January 12, 2024)

eTable 2. Inclusion and Exclusion Criteria for Selection of Relevant Studies

eTable 3. Excluded Studies, With Reasons for Exclusion

eTable 4. Critical Appraisal With Criteria From the Joanna Briggs Institute Meta-Analysis of Statistics Assessment and Review Instrument (JBI-MAStARI) for Case Reports

This supplementary material has been provided by the authors to give readers additional information about their work.

© 2025 Heller C et al. *JAMA Network Open*.

eTable 1. Search Strategy for PubMed, Web of Science, and PsycInfo With Number of Hits (January 12, 2024)

Search Strategy for PubMed

	Search	Number of hits
#1	"Brain Injuries, Traumatic"[MeSH Terms]	25,372
#2	"traumatic brain injury"[Title/Abstract] OR "traumatic brain injur*"[Title/Abstract] OR "TBI"[Title/Abstract] OR "head injur*"[Title/Abstract] OR "brain injur*"[Title/Abstract] OR "craniocerebral trauma*"[Title/Abstract] OR "closed head injur*"[Title/Abstract] OR "brain trauma*"[Title/Abstract] OR "intracranial injur*"[Title/Abstract] OR "neurotrauma*"[Title/Abstract] OR "neurological trauma*"[Title/Abstract] OR "head trauma*"[Title/Abstract] OR "skull injury"[Title/Abstract] OR "fractured skull"[Title/Abstract] OR "cerebral trauma*"[Title/Abstract] OR "cranial trauma*"[Title/Abstract] OR "brain damage*"[Title/Abstract] OR "concuss*"[Title/Abstract] OR "blunt force trauma*"[Title/Abstract]	156,926
#3	#1 OR #2	158,925
#4	"Pregnancy"[MeSH Terms]	1,020,048
#5	"pregnancy"[Title/Abstract] OR "pregnan*"[Title/Abstract] OR "gestation"[Title/Abstract] OR "antenatal peri*"[Title/Abstract] OR "obstetric complicat*"[Title/Abstract] OR "perinatal peri*"[Title/Abstract] OR "gestational peri*"[Title/Abstract] OR "obstetric care"[Title/Abstract] OR "maternal care"[Title/Abstract] OR "maternal health"[Title/Abstract] "prenatal health"[Title/Abstract] OR "perinatal health"[Title/Abstract] OR "gestational health"[Title/Abstract] OR "pregnancy complicat*"[Title/Abstract] OR "pregnancy outcome*"[Title/Abstract] OR "obstetric outcome*"[Title/Abstract] OR "perinatal outcome*"[Title/Abstract] OR "fetal health"[Title/Abstract] OR "obstetric procedure*"[Title/Abstract]	71,401
#6	#4 OR #5	1,030,576
#7	#3 AND #6	2,946
#8	#3 AND #6 Filters: English	2,644
#9	#3 AND #6 Filters: English, German	2,743
#10	#3 AND #6 Filters: English, German, Spanish	2,770
#11	#3 AND #6 Filters: English, German, Spanish, Humans	2,044
#12	("Meta-analysis"[Publication Type] OR "Systematic Review"[Publication Type] OR "Review"[Publication Type])	3,443,975
#13	#11 NOT #12	1,462
#14	#11 NOT #12 Filters: from 1990 - 2023	1,207

© 2025 Heller C et al. *JAMA Network Open*.

Search Strategy for Web of Science

	Search	Number of hits
#1	TI=("traumatic brain injury" OR "traumatic brain injur*" OR TBI OR "head injur*" OR "brain injur*" OR "craniocerebral traum*" OR "closed head injur*" OR "brain trauma*" OR "intracranial injur*" OR neurotrauma* OR "neurological trauma*" OR "head trauma*" OR "skull injur*" OR "fractured skull" OR "cerebral trauma*" OR "cranial trauma*" OR "brain damage*" OR concuss* OR "blunt force trauma*")	114,918
#2	AB=("traumatic brain injury" OR "traumatic brain injur*" OR TBI OR "head injur*" OR "brain injur*" OR "craniocerebral traum*" OR "closed head injur*" OR "brain trauma*" OR "intracranial injur*" OR neurotrauma* OR "neurological trauma*" OR "head trauma*" OR "skull injur*" OR "fractured skull" OR "cerebral trauma*" OR "cranial trauma*" OR "brain damage*" OR concuss* OR "blunt force trauma*")	162,307
#3	AK=("traumatic brain injury" OR "traumatic brain injur*" OR TBI OR "head injur*" OR "brain injur*" OR "craniocerebral traum*" OR "closed head injur*" OR "brain trauma*" OR "intracranial injur*" OR neurotrauma* OR "neurological trauma*" OR "head trauma*" OR "skull injur*" OR "fractured skull" OR "cerebral trauma*" OR "cranial trauma*" OR "brain damage*" OR concuss* OR "blunt force trauma*")	57,460
#4	#1 OR #2 OR #3	216,971
#5	TI=(pregnancy OR pregnan* OR gestation OR "antenatal peri*" OR "obstetric complicat*" OR "perinatal peri*" OR "gestational peri*" OR "obstetric care" OR "maternal care" OR "maternal health" OR "prenatal health" OR "perinatal health" OR "gestational health" OR "pregnancy complicat*" OR "pregnancy outcome*" OR "obstetric outcome*" OR "perinatal outcome*" OR "fetal health" OR "obstetric procedure*")	409,149
#6	AB=(pregnancy OR pregnan* OR gestation OR "antenatal peri*" OR "obstetric complicat*" OR "perinatal peri*" OR "gestational peri*" OR "obstetric care" OR "maternal care" OR "maternal health" OR "prenatal health" OR "perinatal health" OR "gestational health" OR "pregnancy complicat*" OR "pregnancy outcome*" OR "obstetric outcome*" OR "perinatal outcome*" OR "fetal health" OR "obstetric procedure*")	736,367
#7	AK=(pregnancy OR pregnan* OR gestation OR "antenatal peri*" OR "obstetric complicat*" OR "perinatal peri*" OR "gestational peri*" OR "obstetric care" OR "maternal care" OR "maternal health" OR "prenatal health" OR "perinatal health" OR "gestational health" OR "pregnancy complicat*" OR "pregnancy outcome*" OR "obstetric outcome*" OR "perinatal outcome*" OR "fetal health" OR "obstetric procedure*")	152,087
#8	#5 OR #6 OR #7	924,516
#9	#4 AND #8	2,961
#10	#4 AND #8 and English or German or Spanish (Languages)	2,803
#11	#4 AND #8 and English or German or Spanish (Languages) and Review Article (Document Types)	512
#12	#10 NOT #11	2,291
#13	(#12) AND PY=(1990-2023)	2,115

© 2025 Heller C et al. *JAMA Network Open*.

Search Strategy for PsycInfo

	Search	Number of hits
S1	TI "traumatic brain injury" OR "traumatic brain injur*" OR TBI OR "head injur*" OR "brain injur*" OR "craniocerebral traum*" OR "closed head injur*" OR "brain trauma*" OR "intracranial injur*" OR neurotrauma* OR "neurological trauma*" OR "head trauma*" OR "skull injur*" OR "fractured skull" OR "cerebral trauma*" OR "cranial trauma*" OR "brain damage*" OR concuss* OR "blunt force trauma*"	27,107
S2	AB "traumatic brain injury" OR "traumatic brain injur*" OR TBI OR "head injur*" OR "brain injur*" OR "craniocerebral traum*" OR "closed head injur*" OR "brain trauma*" OR "intracranial injur*" OR neurotrauma* OR "neurological trauma*" OR "head trauma*" OR "skull injur*" OR "fractured skull" OR "cerebral trauma*" OR "cranial trauma*" OR "brain damage*" OR concuss* OR "blunt force trauma*"	49,560
S3	KW "traumatic brain injury" OR "traumatic brain injur*" OR TBI OR "head injur*" OR "brain injur*" OR "craniocerebral traum*" OR "closed head injur*" OR "brain trauma*" OR "intracranial injur*" OR neurotrauma* OR "neurological trauma*" OR "head trauma*" OR "skull injur*" OR "fractured skull" OR "cerebral trauma*" OR "cranial trauma*" OR "brain damage*" OR concuss* OR "blunt force trauma*"	35,827
S4	S1 OR S2 OR S3	53,874
S5	TI pregnancy OR pregnan* OR gestation OR "antenatal peri*" OR "obstetric complicat*" OR "perinatal peri*" OR "gestational peri*" OR "obstetric care" OR "maternal care" OR "maternal health" OR "prenatal health" OR "perinatal health" OR "gestational health" OR "pregnancy complicat*" OR "pregnancy outcome*" OR "obstetric outcome*" OR "perinatal outcome*" OR "fetal health" OR "obstetric procedure*"	20,689
S6	AB pregnancy OR pregnan* OR gestation OR "antenatal peri*" OR "obstetric complicat*" OR "perinatal peri*" OR "gestational peri*" OR "obstetric care" OR "maternal care" OR "maternal health" OR "prenatal health" OR "perinatal health" OR "gestational health" OR "pregnancy complicat*" OR "pregnancy outcome*" OR "obstetric outcome*" OR "perinatal outcome*" OR "fetal health" OR "obstetric procedure*"	62,449
S7	KW pregnancy OR pregnan* OR gestation OR "antenatal peri*" OR "obstetric complicat*" OR "perinatal peri*" OR "gestational peri*" OR "obstetric care" OR "maternal care" OR "maternal health" OR "prenatal health" OR "perinatal health" OR "gestational health" OR "pregnancy complicat*" OR "pregnancy outcome*" OR "obstetric outcome*" OR "perinatal outcome*" OR "fetal health" OR "obstetric procedure*"	29,876
S8	S5 OR S6 OR S7	66,046
S9	S4 AND S8	458
S10	Limiters – Publication Year: 1990-2023	395

© 2025 Heller C et al. *JAMA Network Open*.

eTable 2. Inclusion and Exclusion Criteria for Selection of Relevant Studies

		Inclusion criteria	Exclusion criteria
Basic study conditions	Full text	Full text available	Full text unavailable
	Publication	Published article	Unpublished article
	Year of publication	Published between 1990 and 2023	Published before 1990 or after December 31st, 2023
	Language	Article in English, German, or Spanish language	Article in a language other than English, German, or Spanish
	Human study	Human study	Animal or non-human study
	Study design	Experimental and epidemiological study designs including randomized controlled trials, non-randomized controlled trials, quasi-experimental studies, longitudinal studies, prospective and retrospective cohort studies, cross-sectional studies, descriptive cross-sectional studies, case reports, case series, peer-reviewed studies with primary data	Studies without primary data including review articles and meta-analyses, not peer-reviewed articles
Participants	Pregnancy	Pregnant individual(s)	Non-pregnant individual(s)
	Traumatic brain injury (TBI)	Diagnosis of any form of traumatic brain injury (TBI) including mild, moderate, severe TBI (according to the International Classification of Diseases) in the pregnant individual(s)	No diagnosis of any form of TBI in the pregnant individual(s), diagnosis of TBI in the fetus / fetal TBI
Outcomes	Maternal outcomes	Sufficient information on maternal outcomes including the severity of TBI, maternal death	No sufficient information on maternal outcomes to perform an analysis
	Fetal outcomes	Sufficient information on fetal outcomes before, during and/or after birth including fetal death, adverse birth outcomes (e.g. premature birth, low birth weight)	No sufficient information on fetal outcomes to perform an analysis

Abbreviation: TBI, traumatic brain injury.

© 2025 Heller C et al. *JAMA Network Open*.

eTable 3. Excluded Studies, With Reasons for Exclusion

Reference	Title	Reason for exclusion
Adams et al. (2019)	Pregnancy and Neonatal Outcomes Among Women with Traumatic Brain Injury	Duplicate
Akdemir et al. (2014)	Intracranial foreign body (bullet) during pregnancy	Gun shot wound 8 years prior to pregnancy and<br>was not removed<br>TBI before pregnancy
Albini et al. (2023)	Intimate Partner Violence and Pregnancy: Nationwide Analysis of Injury Patterns and Risk Factors	No full text
Ali et al. (1997)	Predictors of fetal mortality in pregnant trauma patients	No full text
Anto-Ocrah et al. (2022)	Pregnancy After Concussion: A Clarion Call for Attention?	Concussion before pregnancy<br>TBI before pregnancy
Baethmann et al. (1996)	Fetal CNS damage after exposure to maternal trauma during	Tables confirm mother in MVA, but do not confirm whether or not there was a TBI<br>Focus on fetus<br>Fetal, no maternal TBI
Berry et al. (2012)	Response to: Do pregnant women have improved outcomes after traumatic brain injury?	Comment<br>No original empirical data
Bickethaupt & Neeley (2016)	Poster 365 Traumatic Brain Injury Resulting from Pontine and Extrapontine Myelinolysis due to Acute Onset of Pregnancy Induced Diabetes Insipidus: A Case Report.	Insufficient information
Birchenall et al. (2023)	Pregnancy outcomes following BMT ± TBI: A prospective UKOSS observational study	BMT ± total body irradiation (TBI)<br>No TBI in pregnant patients
Brookfield et al. (2013)	Maternal death in the emergency department from trauma	Trauma in general<br>No seperate analysis of TBI
Burkle et al. (2015)	Medical, legal, and ethical challenges associated with pregnancy and catastrophic brain injury	No original empirical data
Buskard (1995)	Pregnancy after TBI	No full text
Caban et al. (2011)	Open reduction and internal stabilization of acetabular fractures in pregnancy--case report	No full text
Carvalho et al. (2013)	Anesthetic approach of pregnant woman with cerebral arteriovenous malformation and subarachnoid hemorrhage during pregnancy: Case report.	Excluded after discussion because no TBI was described<br>No TBI in pregnant patients
Colantonio et al. (2010)	Women's health outcomes after traumatic brain injury	Women are premenopausal but not pregnant at time of injury<br>No TBI in pregnant patients

© 2025 Heller C et al. *JAMA Network Open*.

Reference	Title	Reason for exclusion
Eastes (2023)	Neuroendocrine healing after TBI during pregnancy	No full text
Emick-Herring & Mahoney (1994)	Traumatic brain injury, pregnancy, and related management: a case study.	No full text
Fildes et al. (1992)	Tauma - The leading cause of maternal death	Focus on death due to trauma<br>No TBI in pregnant patients
Fins et al. (2013)	Surrogate decision making in the case of a pregnant woman newly disabled with brain injury.	Insufficient information
Fins (2013)	Traumatic Brain Injury and the Pregnant Pause	Comment<br>No original empirical data
Gomez-Rhenals et al. (2021)	Management of Traumatic Brain Injury in Pregnancy: Simultaneous Craniectomy and Cesarean Section Surgeries	No original empirical data
Greenblatt et al. (1997)	Incidence of hospitalized injuries among pregnant women in Maryland, 1979-1990	Hospitalized injuries, trauma in general<br>No seperate analysis of TBI
Hammond (2013)	Moving Beyond Risks Versus Benefits: Pregnancy Options Counseling In A Patient with Traumatic Brain Injury	No full text
Hayes et al. (2007)	Cerebral palsy after maternal trauma in pregnancy	Maternal trauma in general<br>No separate analysis of TBI
Karimi et al. (2004)	Extensive brain injury in a premature infant following a relatively minor maternal motor vehicle accident with airbag deployment	Fetal brain injury<br>Fetal, no maternal TBI
Keneally et al. (2021)	Trauma in obstetrical patients	Trauma in general<br>No seperate analysis of TBI
Kerns (1990)	Unborn children	Book section<br>No (original) empirical data
Kho & Abdullah (2018)	Management of Severe Traumatic Brain Injury in Pregnancy: A Body with Two Lives	No (original) empirical data
Knuppel et al. (1994)	Documented fetal brain damage resulting from a motor vehicle accident	Fetal brain damage<br>Fetal, no maternal TBI
Kredel et al. (2021)	Pregnancy and Irreversible Loss of Brain Functions - Case Report	Irreversible loss of brain function<br>No TBI in pregnant patients
Kuczkowski (2004)	Trauma in pregnancy. Perioperative anesthetic considerations for the head-injured pregnant trauma victim.	No (original) empirical data
Lolk et al. (2022)	Perinatal adversities and risk of epilepsy after traumatic brain injury: A Danish nationwide cohort study.	Fetal, no maternal TBI<br>Epilepsy risk after TBI as outcome
Lolk et al. (2021)	Adverse perinatal outcomes and risk of epilepsy after traumatic brain injury: A Danish nationwide cohort study	Fetal, no maternal TBI

(continued)

© 2025 Heller C et al. *JAMA Network Open*.

Reference	Title	Reason for exclusion
Mesdaghinia et al. (2012)	Causes of trauma in pregnant women referred to shabih-khani maternity hospital in kashan	Trauma in pregnancy<br>No separate analysis of TBI
Mut Buigues. (2006)	Brain injury after fetal traumatism due to maternal traffic accident	Fetal, no maternal TBI
Nguyen et al. (2022)	An Injustice to the Justice-Involved: A Brief Report on the Impact of Traumatic Brain Injury<br>on Incarcerated Mothers	No full text
Pietsch et al. (2013)	Trauma treatment of a severely injured pregnant patient. An interdisciplinary challenge for primary and emergency room treatment	Trauma in general during pregnancy<br>No TBI in pregnant individual
Reinhold et al. (2021)	Ethical, Psychosocial and Legal<br>Aspects of the Treatment of Pregnant Patients with Brain Death	Duplicate
Sadro et al. (2012)	Case report: lethal fetal head injury and placental abruption in a pregnant trauma patient	Fetal trauma, focus on abdominal trauma<br>No pregnant individual(s) with TBI
Saliba (2001)	Perinatal brain injury	Editorial, no data<br>Insufficient information
Santi et al. (2011)	P48. Brain injury on pregnancy	Poster? No full data<br>Insufficient information
Shah et al. (1998)	Trauma in pregnancy: Maternal and fetal outcomes	No full text
Thrasher & McAtee (2016)	Collaborative Care for a Woman With Traumatic Brain Injury During Pregnancy	Seems like an abstract or a conference; full data not available<br>Insufficient information
Toescu et al. (2017)	Frontal skull osteoblastoma with aneurysmal bone cyst-like changes associated with trauma<br>during pregnancy: a case report	Influence of pregnancy and<br>trauma on osteoblastoma behavior<br>No pregnancy outcomes
Torres & Lande (2015)	Objective and personalized longitudinal assessment of a pregnant patient with post severe brain trauma	Analytical techniques to process data from physically wearable sensors<br>No pregnancy outcomes
Vaajala et al. (2022)a	Pregnancy and delivery after traumatic brain injury: a nationwide population-based cohort study in Finland	Pregnancy and delivery after TBI<br>TBI before pregnancy
Vaajala et al. (2022)b	Birth rate after major trauma in fertile-aged women: a nationwide population-based cohort study in Finland	Birth rate after major trauma in fertile-aged women<br>No TBI in pregnant patients
Vaajala et al. (2023)c	Multifetal gestations after traumatic brain injury: a nationwide register-based cohort study in Finland	TBI before pregnancy

(continued)

Reference	Title	Reason for exclusion
Vaajala et al. (2023)d	Previous traumatic brain injury is associated with an increased odds for gestational diabetes: a nationwide register-based cohort study in finland	Previous TBI<br>TBI before pregnancy
Van der Knoop et al. (2015)	Effect of (minor or major) maternal trauma on fetal motility: A prospective study	Excluded after discussion<br>Trauma during pregnancy in general<br>No separate analysis of TBI
Viljoen (1995)	Porencephaly and transverse limb defects following severe maternal trauma in early pregnancy	No full text
Wawrzyniak (2015)	Continuation of pregnancy in a woman with critical brain injury.	No maternal TBI<br>Not only TBI in pregnant individual
Wright et al. (2012)	Response to: Do pregnant women have improved outcomes after traumatic brain injury?	Response<br>No original empirical data
Yamamoto et al. (1999)	A patient with cerebral palsy whose mother had a traffic accident during pregnancy: a diffuse axonal injury?	No maternal TBI<br>No TBI in pregnant patients
Tenami et al. (2023)	The impact of minor trauma during pregnancy on maternal and neonatal outcomes: A tertiary centre experience	Trauma during pregnancy in general<br>No separate analysis of TBI

Abbreviations: TBI, traumatic brain injury

© 2025 Heller C et al. *JAMA Network Open*.

eTable 4. Critical Appraisal With Criteria From the Joanna Briggs Institute Meta-Analysis of Statistics Assessment and Review Instrument (JBI-MAStARI) for Case Reports

**Criteria for case reports**	**Alley et al. (2003)**	**Anquist et al. (1994)**	**Chen et al. (2005)**	**Darlan et al. (2021) Case 1**	**Darlan et al. (2021) Case 2**	**Dawar et al. (2013)**
(1) Were patient's demographic characteristics clearly described?	Yes	Yes	Yes	Yes	Yes	Yes
(2) Was the patient's history clearly described and presented as a timeline?	No	Unclear	Yes	No	No	No
(3) Was the current clinical condition of the patient on presentation clearly described?	Yes	Yes	Yes	Yes	Yes	Yes
(4) Were diagnostic tests or assessment methods and the results clearly described?	Yes	Yes	Yes	Yes	Yes	Yes
(5) Was the intervention(s) or treatment procedure(s) clearly described?	Yes	Unclear	Yes	Unclear	Unclear	Yes
(6) Was the post-intervention clinical condition clearly described?	Unclear	No	Yes	Yes	No	Yes
(7) Were adverse events (harms) or unanticipated events identified and described?	Yes	Yes	Unclear	Yes	Yes	Unclear
(8) Does the case report provide takeaway lessons?	Yes	Yes	Yes	Yes	Yes	Yes
**Final score (/8)**	**6**	**5**	**7**	**6**	**5**	**6**

(continued)

© 2025 Heller C et al. *JAMA Network Open*.

Critical Appraisal With Criteria From JBI-MAStARI for Case Reports

**Criteria for case reports**	**Hnat et al. (2003)**	**Inoue et al. (2016)**	**Neville et al. (2012)**	**Tawfik et al. (2015)**	**Tran et al. (2021)**
(1) Were patient's demographic characteristics clearly described?	Yes	Yes	Yes	Yes	Yes
(2) Was the patient's history clearly described and presented as a timeline?	No	No	Unclear	Yes	Unclear
(3) Was the current clinical condition of the patient on presentation clearly described?	Yes	Yes	Yes	Yes	Yes
(4) Were diagnostic tests or assessment methods and the results clearly described?	Yes	Yes	Yes	Yes	Yes
(5) Was the intervention(s) or treatment procedure(s) clearly described?	Unclear	Yes	Yes	Yes	Yes
(6) Was the post-intervention clinical condition clearly described?	Yes	Yes	Yes	Yes	Yes
(7) Were adverse events (harms) or unanticipated events identified and described?	Yes	Yes	Yes	Yes	Yes
(8) Does the case report provide takeaway lessons?	Yes	Yes	Yes	Yes	Yes
**Final score (/8)**	**6**	**7**	**7**	**8**	**7**

© 2025 Heller C et al. *JAMA Network Open*.

Critical Appraisal With Criteria From JBI-MAStARI for Case Series

**Criteria for case series**	**Kissinger et al. (1991)**	**Leroy-Malherbe et al. (2006)**
(1) Were there clear criteria for inclusion in the case series?	Yes	Yes
(2) Was the condition measured in a standard, reliable way for all participants included in the case series?	Yes	Yes
(3) Were valid methods used for identification of the condition for all participants included in the case series?	Yes	Yes
(4) Did the case series have consecutive inclusion of participants?	Yes	No
(5) Did the case series have complete inclusion of participants?	Yes	No
(6) Was there clear reporting of the demographics of the participants in the study?	Yes	Yes
(7) Was there clear reporting of clinical information of the participants?	Yes	Yes
(8) Were the outcomes or follow up results of cases clearly reported?	Yes	Yes
(9) Was there clear reporting of the presenting site(s)/clinic(s) demographic information?	Yes	No
(10) Was statistical analysis appropriate?	Yes	No
**Final score (/10)**	**10**	**6**

© 2025 Heller C et al. *JAMA Network Open*.

Critical Appraisal With Criteria From JBI-MAStARI for Cohort Studies

**Criteria for cohort studies**	**Adams et al. (2023)**	**Berry et al. (2011)**	**Ganesh et al. (2022)**	**Vaajala et al. (2023)**
(1) Were the two groups similar and recruited from the same population?	Yes	Yes	Yes	Yes
(2) Were the exposures measured similarly to assign people to both exposed and unexposed groups?	Yes	Yes	Yes	Yes
(3) Was the exposure measured in a valid and reliable way?	Unclear	Yes	Unclear	Unclear
(4) Were confounding factors identified?	Yes	Yes	No	Yes
(5) Were strategies to deal with confounding factors stated?	Yes	Yes	n/a	Yes
(6) Were the groups/participants free of the outcome at the start of the study (or at the moment of exposure)?	Yes	Yes	n/a	Yes
(7) Were the outcomes measured in a valid and reliable way?	Yes	Yes	Yes	Yes
(8) Was the follow up time reported and sufficient to be long enough for outcomes to occur?	No	No	Yes	No
(9) Was follow up complete, and if not, were the reasons to loss to follow up described and explored?	No	No	Yes	No
(10) Were strategies to address incomplete follow up utilized?	Unclear	Unclear	Unclear	n/a
(11) Was appropriate statistical analysis used?	Yes	Yes	Yes	Yes
**Final score (/11)**	**7**	**8**	**6**	**7**

Abbreviation: n/a, not applicable.

© 2025 Heller C et al. *JAMA Network Open*.
