# Supplementary material for: Complications After Maternal Traumatic Brain Injury During Pregnancy: A Systematic Review
Source: JAMA Netw Open. 2025 Feb 17;8(2):e2459877. doi: 10.1001/jamanetworkopen.2024.59877 (PMC11833521; doi:10.1001/jamanetworkopen.2024.59877)
Supplement: Supplement 2. — Data Sharing Statement [file jamanetwopen-e2459877-s002.pdf]

Data Sharing Statement

Heller. Complications After Maternal Traumatic Brain Injury During Pregnancy. *JAMA Netw Open*. Published February 17, 2025. doi:10.1001/jamanetworkopen.2024.59877

Data
Data available: No

Additional Information
Explanation for why data not available: It is a systematic review, data is already openly available.
